# Access to treatment in chronic kidney disease, dialysis and transplantation. Is there gender equality?

**DOI:** 10.3389/fmed.2023.1176975

**Published:** 2023-06-21

**Authors:** Jihan Sleiman, Gervasio Soler Pujol, Erika Montañez, Veronica Roatta, Gustavo Laham

**Affiliations:** Internal Medicine Department, Nephrology Section, Centro de Educación Médica e Investigaciones Clínicas Norberto Quirno (CEMIC), Buenos Aires, Argentina

**Keywords:** gender, sex, equality, Chronic kidney disease, dialysis (ESKD), kidney transplantation

## Abstract

Sex and gender are often used as synonyms. However, while sex describes only a biological state, gender is a dynamic concept that takes into account psychosocial and cultural aspects of human existence that can change according to place and time. Inequality in medicine has been described in several areas. Among them, gender inequality has been disregarded for many years and is now a matter of concern. Chronic kidney disease (CKD) is a growing epidemic worldwide, affecting approximately 10% of the population. Although both men and women are affected, gender equality, especially in access to different treatments, is a matter of concern. We decided to investigate gender equality in patients with CKD. To this end, we conducted a literature narrative review to determine whether gender inequalities were found in CKD patients in general and in access to different treatment modalities in particular. A non-language restricted search was performed until November 30th 2022 in PubMed, SciELO, Trip Database, Google Scholar, MEDES y MEDLINE. We also investigated the situation in this regard in our country. We found that CKD is more prevalent in women than men, nevertheless this prevalence decreases along the CKD stages to the point that more men reach end stage kidney disease (ESKD) and dialysis. Access to transplant (ATT) is higher in men than in women although posttransplant survival shows no gender differences. Finally, most series have shown that women are more frequently Kidney transplantation (KT) living donors than men. Results in our country are similar to the published literature with the exception of a higher proportion of men as KT living donors. As in other areas, gender inequality in Nephrology has been largely overlooked. In this review we have highlighted gender differences in CKD patients. Gender inequality in Nephrology exists and needs to be looked upon in order to reach a personalized clinical approach.

## Introduction

Sex and gender are many times used as synonyms. Nevertheless, while sex addresses only a biological condition, gender is a dynamic concept addressing psychosocial and cultural aspects of the human condition that might change with place and time (1). Gender inequality is a very trendy topic that is being discussed in many areas including medicine.

Inequality in medicine has been described in several areas. In particular, gender inequality, which was overlooked for many years, is now an issue of concern. For example, in coronary artery disease, the leading cause of death for both men and women, some studies show that the former are treated more aggressively than the latter, who even have longer wait times at emergency room (2).

Chronic Kidney disease’s (CKD) prevalence increases annually affecting 11.8% of women and 10.4% of men worldwide, according to recent data from the International Society of Nephrology (ISN) (1). Of the various causes of CKD, Diabetes mellitus (DM) and Hypertension (HTN) account for two third of the cases. DM has been identified as the first cause in most developed economies, reaching 44% of the cases in the USA. In those patients reaching end stage kidney disease (ESKD), preemptive Kidney transplantation (KT), whenever possible, is the treatment of choice (3).

Gender equality in Nephrology has not been a priority. Many research studies in the field account for gender inequality in different areas of nephrology. We wondered if men and women with ESKD have equal access to therapies either dialysis or KT.

Our aim was to investigate gender equality in patients with CKD. We conducted a literature search to determine whether gender inequalities have been reported in CKD patients in general and their access to different treatment modalities in particular. We also investigated the situation in our country in this regard.

## Methods

A non-language restricted search was performed until November 30th 2022 in PubMed, SciELO, Trip Database, Google Scholar, MEDES y MEDLINE, using the following MeSH terms and keywords: “Sex,” “gender” “Inequality,” “dialysis,” “CKD,” “Kidney Transplantation,” “pretransplant” “living kidney donors,” “chronic kidney disease,” “renal replacement therapy,” “renal transplant” “frailty,” “disparity.” Relevant articles and reviews were manually selected by two independent reviewers according to the aforementioned terms. Articles addressing only women or men, non-control studies, pediatric studies and congress abstract were excluded.

## Gender and chronic kidney disease

Gender disparities in CKD have been reported worldwide, although the reasons for this have not been completely clarified. In National health and Nutrition Examination Survey 2015–2018 (NHANES) study, which included CKD patients not on dialysis, there were globally more women than men (16.8 vs. 13.3%). Nevertheless, this women’s predominant prevalence decreased along the CKD stages, in stage III (7.33 vs. 5.47%), stage IV (0.45 vs. 0.3%) and in stage V men were more prevalent than women (0.07 vs. 0.15%) (4). Although there is no clear explanation for the higher prevalence of women in the early stages of CKD, a higher life expectancy and over estimation of glomerular filtration (eGFR) rate in women have been proposed (5). There are also gender differences in terms of CKD etiology. DM, HTN, tobacco abuse, atherosclerotic disease and cancer are mainly reported in men while in women, autoimmune diseases take the first place (6).

Neugarten et al. (7) published a meta-analysis where they included 68 cohort studies to evaluate the risk of progression to ESKD. They showed that men had a higher risk of progression than women. Estrogens may have renal protective effects though a higher production of endothelial nitric oxide synthase, a down regulation of renin-angiotensin system as well as endothelin and NADPH with antiapoptotic and antifibrotic renal effects. These effects, and a higher prevalence of less healthy. lifestyle reported in men could explain the observed differences in renal disease progression (8). Also, the Dialysis Outcomes and Practice Patterns Study (DOPPS) study, performed in developed economies between 1996 and 2012, found a higher prevalence of CKD in women but a higher proportion of men reaching dialysis (9). Acute kidney injury is known to increase the risk of CKD and ESKD. Gender differences in renal anatomy, physiology and risk of renal disease is supported by clinical and experimental data. There is sufficient evidence supporting that gender differences in the risk of developing CKD from AKI are based on differences on renal anatomy and physiology, such as renal tubule’s absorptive and secretory capacity between men and women. There seems to be a protective effect of estrogens in women’s susceptibility to acute kidney damage and their ability to restore residual kidney function. On the other hand, male hormones have demonstrated a detrimental effect but the molecular mechanisms involved are not clear. Also, the evidence supporting gender differences in the repair and replication processes of damaged renal cells affecting males needs to be further investigated (10, 11).

**FIGURE 1:**
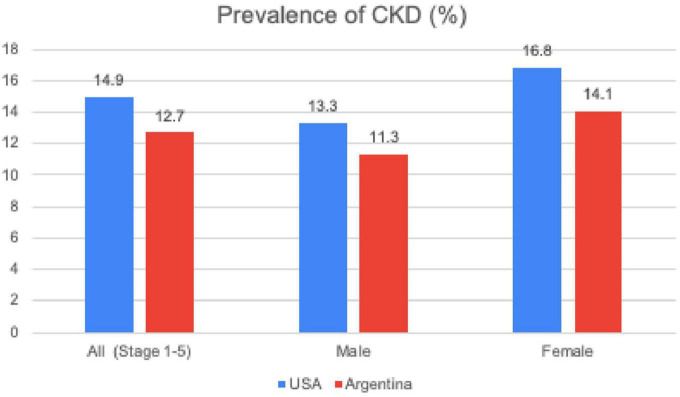
Prevalence of chronic kidney disease. CKD, Chronic Kidney Disease; NHANES, National Health and Nutrition Examination Survey (USA 2015-2018); ENNyS2, Segunda Encuesta Nacional de Salud y Nutricion de Argentina (2019).

In Argentina According to the second national health and nutrition survey performed between 2018 and 2019, the prevalence of CKD was 12.7%. Stages IIIa and IIIb were the most frequent with 6.8 and 1.9%, respectively. CKD was more prevalent in women for all stages (12; Figure 1).

## Gender and dialysis

Gender disparity is also evident in patients under renal replacement therapy. Women in the general population have a survival advantage over men, but this advantage is lost once they reach ESKD. Moreover, women who start dialysis have a higher risk of hospital readmissions in the first 30 days (13). Also In DOPPS study, women started dialysis with lower eGFR than men (10.1 vs. 10.7 ml/min) and older age (9). The reasons for these findings are unclear. Women in general have lower muscle mass, less protein consumption and higher reduction of body mass especially after menopause than men, this could result in a lower creatinine value at the same level of eGFR leading to a later dialysis initiation and higher mortality (14).

### Vascular access

Many series report that the percentage of women dialyzing through a native AVF are less than men. Also, the life span of native AVF in women is reduced even under standardized vascular mapping. A European multicenter study involving 1247 patients found that native AVF was present in 73% of women and 80% of men (15). Also, in Spain a study reported that 20.8% of women are dialyzed through a catheter, 67.7% through a native AVF and 11.5% through an AV graft vs. 10.8, 81.2, and 8%, respectively in men. This gender disparity in the vascular access could be related to gender differences in the size and quality of vessels leading to a higher obstruction rate and a higher incidence of infectious complications reported in women (16).

### Dialysis dose

Women have a higher percentage of fat tissue than men therefore Calculated Volume (V) and the V/BSA ratio are lower. This may lead to an overestimation of Kt/V in this population, which tends to dialyze less than men with the same Kt/V goal (17). On the other hand, women have a greater high metabolic rate compartment (HMRC) resulting in a higher production of uremic toxins. Dialysis dose based on calculated V may result in under estimation of the time needed to eliminate middle size molecules, phosphorus and protein bound substances in women. The loss of women’s survival advantage over men when reaching ESKD could be explained by some of these factors (14).

A study performed by Sridharan et al. (18) in the UK in 2012 which included 1,500 patients on HD, found that women require a higher dialysis dose to improve survival compared to men (Kt/K sp 1.6 vs. 1.4). Also another European study involving 1,247 patients found that dialysis dose was higher in women (Kt/Vsp 1.9 vs. 1.8 and URR 79 vs. 77%) where no differences were found in age, time on dialysis, BMI, Charlson comorbidity index score and proportion of patients with DM or HTN between men and women (15). In a prespecified subgroup analysis of National Institutes of Health-sponsored Hemodialysis (HEMO) study, women randomized to a higher urea clearance had 19% less mortality associated with cardiovascular disease (CVD), cerebrovascular accident (CVA) and vascular access complications, compared to those assigned to a lower clearance (19). These results have been confirmed by the Japanese DOPPS study with a cohort of 5,784 hemodialysis patients, where they found an association between a low urea clearance and increased risk of death, especially in women (20).

Although there is no clear explanation for these findings, the aforementioned women’s higher urea generation rate, an increased sensitivity to uremic toxins or other unknown factors have been proposed. In any case and independent of body size, women seem to benefit from a higher dialysis dose than men (19).

### Dialysis in Argentina

According to the Chronic Dialysis Argentinian Registry, by January 2023 there were 30,072 patients on dialysis of which 93.3% were on hemodialysis. Mean age at dialysis initiation was 59.1 years. Adjusted prevalence was 650 patients per million population (PMP) where 376 PMP were men and 274 PMP were women. Incidence was 161 PMP of which 97 PMP were men and 65 were women. This higher prevalence and incidence of men over women with ESRD was persistent for all age groups and also was consistent with results published by other registries around the globe. Finally, the mortality rate for this population was 17.23% with no differences between men and women 17.8 vs. 16.35%, respectively (21).

## Access to transplantation

Kidney transplantation waitlist (WL) inclusion process starts with patient referral to a Transplant center where all the information concerning risks and benefits of KT will be given. With the patient’s consent, a complete pre transplant evaluation will be done to determine the patient’s aptitude for the procedure. This complex process has several steps that sometimes-become pitfalls for the final inclusion on the WL (22).

Several studies have shown that women with ESKD have at least 10 to 20% less access to Kidney transplantation (ATT) than men after adjusting for the main demographic and clinical variables. United States Renal Data System (USRDS) and United Network of Organ Sharing (UNOS) data showed that over 563,197 patients started on dialysis between 2000 and 2005, 14% were included on the WL. Women had 11% less ATT than men. Age adjusted data showed that this difference increased with age. Men and Women between 18 and 45 years old had equal ATT, while women aged 66 to 75 had 29% less ATT from deceased donor and 30% from a living donor (RR 0.70; IC del 95%: 0.63 a 0.78; *P* < 0,001). This difference persisted after adjusting for comorbidities such as DM, CVD or CVA (23). Another study from the same source found that women have 21% higher chances of being reported as unsuitable for KT because of their age, 5% higher chances of being declared medically unfit (after adjusting for clinical and demographic data) and 23% higher chances of consent decline or reporting lack of information about KT, than men (24). These findings have been confirmed in a more recent study with over 45,000 incident dialysis patients between 2012 and 2016, where women had 14% less chance of referral for KT than men. As in the previous studies, this difference persisted after adjusting for demographic and clinical data and increased with age (6). Finally a Canadian study over 13.000 patients also found in an adjusted multivariate analysis that women had 12% less ATT than men (25).

Social limitations for ATT in women have been reinforced by some studies. Salter et al. found that women had 45% less probability of discussing KT with a transplant physician (26). Women could be reluctant to KT due to a higher concern (72 vs. 55%) about medical and psychosocial complication of KT than men (27).

As we mentioned many patients struggle to progress through the different steps of the WL process. Alexadre CG et al. evaluated 4,597 incident patients on dialysis and their likelihood of progressing, going backwards or dying through the following steps: (A) being medically suitable and possibly interested in transplantation, (B) being definitely interested, (C) completing the pre-transplantation workup, and (D) moving up a waiting list and receiving a transplant. The probability of staying stationary in sept A was 78, B 82, C 81, and D 90%, backward movement was rare ranging 3 to 7%, death ranged from 7 to 22%. Women had higher risk of dying in step C compared to men (15 versus 11%; *p* < 0.04) (28).

According to the Organ Procurement and Transplantation Network (OPNT), by 2021 women represented only 38% of the WL. Data from USRDS showed by 2013 that time on the WL was 47.7% in men and 49.4% in women. This difference persists along the WL of different organs (4). In Argentina up to 1/1/23 18% (*n* = 5,407) of the patients older than 18 years old in dialysis were on WL, 45.4% (*n* = 2,456) were women vs. 54,6% (*n* = 2,951) men (21). In summary, ATT is lower in women than in men. This gender disparity increases with age and comorbidities. It could be explained in part by differences in perceptions on women’s ability to benefit from KT compared to men. Aged and comorbid women may see themselves, or be seen by others as more fragile or incapable of coping with transplant surgery than men of the same age and characteristics. We will discuss more about this in the next section.

## Gender and transplantation

Consistent with gender disparity on the wait listing, KT also shows differences between sexes. UNOS and the European Transplant Registry report over a period of more than ten years, that approximately 60% of KT recipients are men. Even in more inclusive health care systems such as the French one, this difference persists (29). The DOPPS study also found that ATT was 5.6% in women and 7% in men (*p* < 0.05) (9). There are several explanations for these findings. As discussed in the previous section there are a number of social constraints that limit women’s access to KT (24, 25) as well as medical limitations such as a higher panel reactive antibody (PRA) or active autoimmune diseases reported in women (29). Perceived or auto perceived frailty may also lead to women being misclassified as frail, limiting their ATT especially in the elderly (30, 31). A European study reported that women older than 60 years were seen as more fragile than men of the same age. As a result, women between 65 and 75 years had 29% less ATT than their male counterparts and this difference reached 59% in patients older than 75 years. Nevertheless, for those reaching KT, patients and graft survival were similar in men and women (4).

According to the Argentinian procurement and Transplant registry SINTRA, in Argentina the donation rate has been variable in the last decade. After reaching a peak at 19.6 donors PMP in 2019, it went down to 16.6 PMP after the COVID pandemic. Between 2013 and 2022, 8,903 patients have received a KT from deceased donors of whom 42.3 and 41.7% were female recipients and donors, respectively. During the same period, 2,540 patients received a KT from a living donor of whom 43.8 and 46.3% were female recipients and donors, respectively (21).

## Outcome after KT

As mentioned before, sensitization prior to transplantation tends to be higher in women due mainly to previous pregnancies. According to some reports, despite a higher adherence in women, there seems to be also a higher susceptibility to graft rejection due to a greater humoral and cellular response than men (4). Some gender-related biological differences could explain these findings. Approximately 50 immune-related genes are expressed in chromosome X and they could be overexpressed in women. Hormone driven differences in toll-like receptors and dendritic cell differentiation could explain more vigorous cellular and innate cell responses in women. Progesterone and Testosterone favor a Th2 profile while estrogens favor Th1. The latter also favors Lymphocytes B survival and antibody (Ab) production. This could explain a stronger humoral response seen in women. On the contrary testosterone has a negative effect on Lymphocyte B and T survival. After menopause estrogens level reduction together with immunosenescence could explain graft survival improvement reported in this women subgroup (29, 32).

Gender D/R mismatch may impact graft outcomes after KT but studies are inconsistent. Kim and Gill reported in their study that kidneys from female donors have a higher incidence of acute rejection and short-term graft loss. Age and gender adjusted analysis revealed that female organs on male recipients had worse survival and vice versa. Reduced nephron mass, a higher HLA expression were some of the possible explanations (31, 33). On The other hand, another cohort study over 159,417 patients from the “Scientific Registry of Transplant Recipients” (SRTR) found that organs from male donors on female recipients younger than 45 years old, had a higher incidence of graft failure HR 1.14 (95% CI, 1.03–1.26). Over 45 years, the risk persisted but it was reduced to HR 0.95 (95% CI, 0.91–0.99). A decreased immune response associated with postmenopausal hormonal changes, adherence and reduced body mass were mentioned as explanations (34) Finally Sancho et al. (35), in a retrospective study over one thousand patients, found that gender did not have an impact on graft outcomes. Chronic rejection in women and death with functioning graft were main reasons for graft loss.

Immunosuppressive drug’s metabolism is also reported to be different between men and women KT recipients. Calcineurin inhibitor’s clearance could be higher in women, but ABCB1 gene expression and gp-P activity are lower leading to higher intracellular drug levels. Mycophenolic acid’s clearance has been reported to be 10–25% lower in women than men, and finally the expression of six-mercaptopurina’s catabolizing enzyme expression is 14% higher in men than women. Finally, prednisolone metabolism seems to be slower in women which could lead to a higher risk of adverse events (4).

Malignancies are a well-known cause of morbidity and mortality after KT. The risk of many of them is higher than in the general population and associated with the effect of immunosuppression. In young KT recipients the risk is slightly higher in women than men (HR for men 0.9 CI 95% 0.8–0.9) while in elderly patients, men have higher incidence of malignant tumors. Women have higher incidence of Kaposi Sarcoma, lung and gynecological malignancies, while in men bladder and kidney cancer are predominant. There seems to be no gender differences in immunosuppression related malignancies such as PTLD and malignant melanoma. Mortality has been reported to be higher in men (36).

## Gender and living kidney donation

Over the year’s most series have shown that women are more prone to be kidney donors than men. Back from the first registers in Human Transplant Kidney in the 60’s and 70’s, 54% of donors were women (4). In the 1990s over a period 10 years the OPTN reported in the USA over 30.258 living donors KT, 87% were living related and 13% unrelated donors, of the latter, 8% were spouses of which 68% were from women to men (*p* < 0.001) (36). A retrospective study with data from the USRDS, Bloembergen et al., found that women were 10% less chance of receiving a living donor KT than men (*p* < 0.01) and 28% higher chances of being a KT donor than men (37). Recently, a study published with US and Austrian data, has shown for the US cohort that more living donor kidneys originated from women rather than men. This tendency continues today, with 74% wife to husband donation by 2019 (38). According to UNOS Registry during 2022, a total of 5,864 patients received a KT from a living donor of whom 36.2% were female recipient and 63.7% were female donors (39) (Table 1). There are several explanations for these findings such as Socio-Cultural patterns placing women as responsible for “family’s wellbeing” and men for “family’s financial support.” The economic limitation may lead to a situation of cohesion, especially in countries with no legal protection for organ trafficking where women and girls are the main victims of transplant tourism (40, 41). Finally, medical constraints have been mentioned, such as a higher incidence of CV comorbidities in men that would contraindicate donation (4, 40). Nevertheless, this has been argued by others who have found that of those medically accepted for kidney donation, 30% of women and 7% of men gave their consent (42).

**TABLE 1:** Donor gender in living kidney transplantation.

References	Year	Living donors transplant (n)	Recipient gender Male/Female%	Donor gender Male/Female%	Comments
Kayler et al. (36)	1990–1999	30.258	57.7/42.3	42.9/57.1	Same results for related and non-related donors
Bloembergen et al. (37)	1991–1993	5.711	58.5/41.5	43.8/56.2	Women 28% higher probability of being kidney donor than men
OPTN* (39)	2022	5.864	63.8/36.2	36.3/63.7	–
SINTRA* (21)	2013–2022	2.540	56.2/43.8	53.7/46.3	–

*OPTN, https://optn.transplant.hrsa.gov/data/view-data-reports/national-data/. *SINTRA, Sistema Nacional de Información de Procuración y Trasplante de la República Argentina.

Causes of kidney donor discard have also been explored according to gender. In a study performed by Wake Forest University, 541 potential Kidney donors were excluded, of which 315 (58.2%) were women. Suboptimal renal function (7.9 vs. 0.9% *p* < 0.0001) and evaluation not completed 6.4 vs. 1.8% *p* = 0.01) were found to be statistically different between women and men (41). The aforementioned over estimation of eGFR in women could have influenced renal function differences (11).

Finally, whether there is a gender difference on the kidney donor’s renal function outcome after donation has also been a matter of concern. Data from UNOS/OPTN, over a period of 10 years from 1996 through 2006, found that 126 kidney donors entered the KT waiting list. Mean age at donation was 31 years and 35% were women. Although women donated more than men the latter developed more frequently ESKD, 78% of donors on the WL were men (*p* < 0.0001) (43).

In conclusion, gender inequality in Nephrology has been largely overlooked. In this review we have highlighted gender differences in CKD. Although more men reach ESKD, CKD is more prevalent in women suggesting less progression. Once on dialysis women need higher doses to match men’s survival. Women have lower ATT than men especially in the elderly, although their survival is comparable. Finally, most series have shown that women are more frequently KT living donors than men. Results in our country are similar to the published literature with the exception of a higher proportion of men as KT living donors. Gender inequality in Nephrology exists and needs to be looked upon in order to reach a personalized clinical approach.

## Author contributions

All authors contributed equally to the manuscript production and approved the submitted version.
